# Spotted fever group rickettsiae associated with ixodid ticks in wild environment in Southern Italy

**DOI:** 10.1002/mbo3.527

**Published:** 2017-10-18

**Authors:** Donato Antonio Raele, Domenico Galante, Nicola Pugliese, Giovanna La Salandra, Maria Assunta Cafiero

**Affiliations:** ^1^ Istituto Zooprofilattico Sperimentale della Puglia e della Basilicata Foggia Italy

**Keywords:** Italy, LAMP, rickettsiae, tick, tick‐borne rickettsioses

## Abstract

Ixodidae ticks are vectors and reservoirs of several species of rickettsiae, and tick‐borne rickettsioses are reported worldwide. This study was aimed to verify the distribution of spotted fever group rickettsiae associated with ticks in a wild environment, the National Park of Gargano, where there is proximity between wild and domestic animals, and which is within an endemic area for rickettsiosis. Ticks were collected from animals or vegetation, morphologically identified and tested by a PCR targeting the 17kDa gene, and by a loop‐mediated isothermal amplification (LAMP) targeting *ompB* gene. Out of 34 tested tick pools, 2 from *Dermacentor marginatus,* 1 from *Ixodes ricinus,* and 1 from *Rhipicephalus turanicus* resulted positive. Nucleotide sequences of amplicons showed high similarity with sequences from *Rickettsia slovaca*,* Rickettsia raoultii*,* Rickettsia helvetica,* and *Rickettsia felis*. The overall calculated infection rate was 26.19 per 1,000, while it rose up to 107.77 when only *D. marginatus* was considered. The results highlight the association among *Ri. slovaca*,* Ri. raoultii*,* D. marginatus* and wild boars from which infected ticks were collected. Finally, the study shows the low efficacy of the previously described LAMP method for the detection of *Rickettsia* spp., when compared to PCR, making urgent the development of most effective LAMP protocols.

## 1 INTRODUCTION

Bacterial species belonging to the genus *Rickettsia* (*Ri*.) includes obligate intracellular, pleomorphic, gram‐negative bacteria. Based on genomic features, the genus was classified into three major groups, the typhus group (TG), the scrub typhus group (STG), and the spotted fever group (SFG) (Weinert, Werren, Aebi, Stone, & Jiggins, 2009). The latter includes species that may be mainly transmitted by ixodid ticks to vertebrates through the salivary secretions (Brouqui, Parola, Fournier, & Raoult, 2007). Therefore, the incidence of SFG rickettsioses is closely associated with the distribution of the host arthropods that act as vectors and/or reservoirs (Fournier & Raoult, 2009; Weinert et al., 2009), and which may transmit the infection to humans.

In humans, despite some report of asymptomatic cases (Fournier et al., 2003), the tick‐borne rickettsioses (TBR) usually occur with moderate symptoms such as fever, headache, maculopapular rash with purpuric elements, and eschar formation at the site of the tick bite (Oteo & Portillo, 2012; Parola, Paddock, & Raoult, 2005; Parola & Raoult, 2001). Though rarely, fatal cases have also been reported (Oteo & Portillo, 2012).

The disease is currently considered to be mainly diffused in Europe, in particular, along the Mediterranean basin, insomuch that it is historically known as Mediterranean spotted fever (MSF). *Rickettsia conorii* is the agent considered to be mainly associated with MSF. Among Mediterranean countries, Italy has the highest incidence, recording 4,604 cases from 1998 to 2002, with an average of 921 cases per year (1.6 per 100,000 inhabitants, up to 10 per 100,000 inhabitants in endemic regions such as Sardinia) (Ciceroni, Pinto, Ciarrocchi, & Ciervo, 2006; Madeddu et al., 2016).

However, it is reasonable to assume that the overall incidence of TBR is underestimated: considering the high occurrence of mild forms of TBR, and the lack of specificity of most symptoms, it may be easily misdiagnosed or simply not reported (Parola et al., 2005).

In the last decades, the development of molecular techniques has improved the sensitivity of diagnostic techniques by making possible the detection of *Rickettsia* spp. DNA directly from biological samples. Consequently, rickettsioses recently reported from Europe were found to be caused by many SFG species (Oteo & Portillo, 2012). Furthermore, different SFG species, such as *Ri. helvetica*,* Ri. slovaca*,* Ri. massiliae*,* Ri. raoultii*,* Ri. monacensis*,* Ri. felis*,* Ri. africae*,* Ri. sibirica*,* Ri. mongolitimonae*,* Ri. honei*,* Ri. peacockii,* and *Ri. aeschlimannii*, have also been described in several ticks. Most of those reports came from Italy, where ecological and climatic factors may promote the development and the growth of these arthropods during the year (Beninati et al., 2005; Chisu et al., 2013; Giudice et al., 2014; Mancini et al., 2015; Martello et al., 2013; Otranto et al., 2014; Pennisi et al., 2015; Selmi, Martello, Bertolotti, Bisanzio, & Tommasone, 2009).

However, very few up‐to‐date data (among them, Masala et al., 2012 and Mancini et al., 2015) are available about the diffusion in the wild environment of SFG rickettsiae in Mediterranean Europe, as most of studies were targeted to human infections.

In light of those considerations, this research was aimed to evaluate the circulation of SFG rickettsiae in ticks collected from the environment in the National Park of Gargano (Italy), to gather details about the distribution and the natural hosts of known rickettsia species in the natural environment. Beyond their environmental relevance, data from that park may be of wider interest because of the close contacts between wild fauna and domestic animals, mainly ovine, which range over a part of its area.

The research was performed using a PCR‐based amplification technique and a loop‐mediated isothermal amplification (LAMP) method in order to verify the most suitable approach for the investigations from field.

## 2 EXPERIMENTAL PROCEDURES

### 2.1 Tick collection and identification

From July to October 2013, a collection campaign was undertaken to collect ticks in the National Park of Gargano, in the Apulia region, Italy. The location was chosen because it is an environmentally protected area, but whose boundaries are close to agricultural and animal farms, and this granted a good balance between wild natural and anthropic rural environments. The park is also characterized by a high level of biodiversity of vertebrate and invertebrate species living there.

During the campaign, 158 tick specimens were collected. Among those, 110 were manually removed from domestic and wild dead mammals found in field, and sent to the facilities of the Veterinary Entomology section of the Experimental Zooprophylactic Institute of Apulia and Basilicata (Foggia, Italy). Forty‐eight ticks were collected by the dragging method (Rulison et al., 2013) in three different sites of the park during September 2013.

All specimens were placed in vials containing 70% ethanol and identified according to the morphological keys of Manilla (1998) and Estrada‐Peña, Bouattour, Camicas, and Walker (2014). After identification, the ticks were divided into pools by species and host (Table 1) for the molecular analyses.

**Table 1:** Tick specimens and molecular detection of spotted fever group Rickettsia

Tick species	Host	Pools (size/developmental stage)	SFG‐Rickettsia PCR‐positive pools	SFG‐Rickettsia LAMP‐positive pools	Identified Rickettsia species	MLE 95% confidence interval (central value)
*Ixodes ricinus*	Free living	9 (5/nymphs)	1	0	*Rickettsia helvetica*	1.18–94.86 (19.98)
	Wolf	1 (5/adults)	0	0	—	
*Ixodes acuminatus*	Free living	1 (3/nymphs)	0	0	—	—
*Dermacentor marginatus*	Wild boar	2 (4/adults)	2	2	*Rickettsia raoultii* *Rickettsia slovaca*	21.11–330.80 (107.77)
	Sheep	2 (5/adults)	0	0	—	
		1 (2/adults)	0	0	—	
*Rhipicephalus bursa*	Wolf	1 (5/adults)	0	0	—	—
	Sheep	5 (5/adults)	0	0	—	
*Rhipicephalus sanguineus*	Dog	7 (5/adults)	0	0	—	—
		1 (3/adults)	0	0	—	
		1 (2/adults)	0	0	—	
	Sheep	2 (5/adults)	0	0	—	
*Rhipicephalus turanicus*	Sheep	1 (5/adults)	1	0	*Rickettsia felis*	Not enough data available
Total		34 (158)^a^	4	2	—	8.61–61.94 (26.19)

a The total number of ticks is provided parenthetically.

### 2.2 Detection of rickettsia DNA by PCR

Ticks from each pool were crushed by the mean of sterile pestle and mortar, and the homogenates were subjected to total DNA extraction using the DNeasy blood and tissue extraction kit (Qiagen, Milan, Italy) according to the manufacturer's instructions. Five microliter of each DNA solution were used as a template in the amplification reactions. Detection of SFG *Rickettsia* spp. was performed according to Webb, Mitchell, Malloy, Dasch, and Azad (1990), who set up a PCR protocol targeting the 17 kDa outer membrane protein gene using primers 5′‐GCTCTTGCAACTTCTATGTT‐3′ and 5′‐CATTGTTCGTCAGGTTGGCG‐3′. Thermal cycle was as follows: initial denaturation at 95°C for 5 min and 35 cycles of denaturation at 95°C for 30 s, annealing at 57°C for 2 min, elongation at 72°C for 1 min. Whenever the protocol was not enough to discriminate at species level, the PCR protocol described by Roux, Fournier, and Raoult (1996), targeting the *ompA* gene (primers 5′‐ATGGCGAATATTTCTCCAAAA‐3′ and 5′‐GTTCCGTTAATGGCAGCATCT‐3′), was applied. Thermal cycle consisted of an initial denaturation at 95°C followed by 35 cycles of 95°C for 30 s, 46°C for 45 s, and 72°C for 40 s. In both cases, the REDTaq ReadyMix PCR Reaction Mix (Sigma Aldrich, Milan, Italy) was used.

All the gathered PCR products were purified by the mean of the QIAquick Spin PCR Purification kit (Qiagen) according to the manufacturer's instructions, and the nucleotide sequences of both strands were determined by the BigDye Terminator DNA sequencing kit (Thermo Scientific, Milan, Italy). Sequencing primers were the same used for PCR. The nucleotide sequences of amplicons were submitted in GenBank under the accession numbers KY576905‐KY576908 (17 kDa‐protein gene) and KY576909‐KY576910 (*ompA*). The nucleotide sequences were compared with those present in GenBank by Nucleotide BLAST (Johnson et al., 2008) to confirm the identification.

### 2.3 Detection of rickettsia DNA by LAMP

The detection of SFG *Rickettsiae* by LAMP was carried out according to the protocol of Pan, Zhang, Wang, and Liu (2012), based on the amplification of a portion of the *ompB* gene. The sequences of oligonucleotides are provided in Table 2. The protocol was slightly modified using the Isothermal Master Mix with carboxyfluorescein (Optigene, Horsham, UK) and ROX as a passive reference dye. The reactions were carried out at 65°C for 40 min in a StepOne Real‐Time PCR system (Applied Biosystems, Milan, Italy). For each group of LAMP reactions, a sample with nuclease‐free water was added as negative control; and a reaction with DNA of *Ri. conorii* (kindly provided by the Unité des Rickettsies, Marseille, France) was set up as positive control.

**Table 2:** *In silico* analysis of the annealing of the LAMP primers to their target region within the *ompB* gene of the rickettsia chromosome

Primer	Sequence 5′–3′	Target species		
		Optimal annealing^a^	Acceptable annealing^b^	Uneffective annealing^c^
F1c	GTCACCGCAACATTTGCATCTG	*Ri. raoultii* *Ri. slovaca*		*Ri. felis* *Ri. helvetica*
F2	GTAACACTGCAGGTGTGAT	*Ri. raoultii* *Ri. slovaca*	*Ri. felis*	*Ri. helvetica*
B1c	TACAGCAATTGAAGCATCAGGT	*Ri. raoultii* *Ri. slovaca*		*Ri. felis* *Ri. helvetica*
B2	TCCTAAACGTAACTCGGC	*Ri. raoultii* *Ri. slovaca*		*Ri. felis* *Ri. helvetica*
F3	AGGTGATGCTAIIAATCC	*Ri. raoultii* *Ri. slovaca*		*Ri. felis* *Ri. helvetica*
B3	CTGTACCITCAGCAAGTT	*Ri. raoultii* *Ri. slovaca*		*Ri. felis* *Ri. helvetica*

a 100% identity with the target region.

b No more than three mismatches, and none within the five nucleotides at 3′ end.

c More than three mismatches or one or more mismatches within the five nucleotides at 3′ end.

### 2.4 Analysis of LAMP primers

In order to verify the annealing of the LAMP primers, the sequences of the *ompB* gene from *Ri. helvetica* (GenBank accession number KP866151), *Ri. felis* (CP000053), *Ri. raoultii* (CP019435), and *Ri. slovaca* (CP003375) were aligned by the clustalW algorithm implemented in CLC sequence viewer 7.7.1 (Qiagen, Aarhus, Denmark). The match of the six oligonucleotide sequences with their target was manually checked.

### 2.5 Infection rate calculation

The maximum likelihood estimation (MLE) of the infection rate (IR) and the respective 95% confidence interval were calculated using the PooledInfRate software (Biggerstaff, 2005). Infection rate has been expressed as number of infected ticks per 1,000.

## 3 RESULTS

### 3.1 Tick identification

Following identification, the 158 collected ticks were assigned to six species. Specifically, 50 (31.64%) were identified as *Ixodes* (*I*.) *ricinus,* 3 (1.89%) as *I. acuminatus*, 50 (31.64%) as *Rhipicephalus* (*Rh*.) *sanguineus,* 30 (18.98%) as *Rh. bursa*, 5 (3.16%) as *Rh. turanicus,* and 20 (12.65%) as *Dermacentor* (*D*.) *marginatus* (Table 1).

### 3.2 Detection of rickettsiae in ticks by PCR

Out of the 34 pools of ticks, 4 (11.76%) resulted positive to PCR, returning the expected amplicons. Among positive pools, one consisted of free‐living *I. ricinus*, two of *D. marginatus* (collected from wild boar), and one of *Rh. turanicus* (from a sheep) (Table 1). The BLAST analysis showed that the nucleotide sequence of the amplicon from *I. ricinus* pool was 100% identical to the corresponding region of the NCBI reference sequence of the *Ri. helvetica* chromosome (Accession number NZ_CM001467). The nucleotide sequence of the amplicon from the *Rh. turanicus* pool was 100% identical, to the corresponding region of the *Ri. felis* chromosome (RefSeq accession number NC_007109), which harbored the gene encoding the 17kDa protein. The nucleotide sequence of one amplicon from the *D. marginatus* pool was 99% identical to the corresponding portion of the17kDa gene of reference genomes from several *Rickettsia* species, such as *Ri. raoultii* (accession number NZ_CP010969), *Ri. parkeri* (NC_017044), and *Ri. philipii* (NC_016930), while the other was 100% identical to the corresponding region of *Ri. slovaca* reference genomes (accession numbers NC_017065 and NC_016639), and 99% similar to the reference genomes of several species, such as *Ri. rickettsii* (NZ_CP006009), and *Ri. philipii* (NC_016930). To unequivocally identify the species from the two *D. marginatus* pools, the *ompA* analysis was performed and it revealed that the nucleotide sequence of the amplicon from the first pool was 100% identical to the corresponding region of the *Ri. raoultii* reference genome, and the second was 100% identical to corresponding region of the reference genomes of *Ri. slovaca*.

### 3.3 Detection of rickettsiae in ticks by LAMP/Analysis of LAMP primers

**Figure 1:**
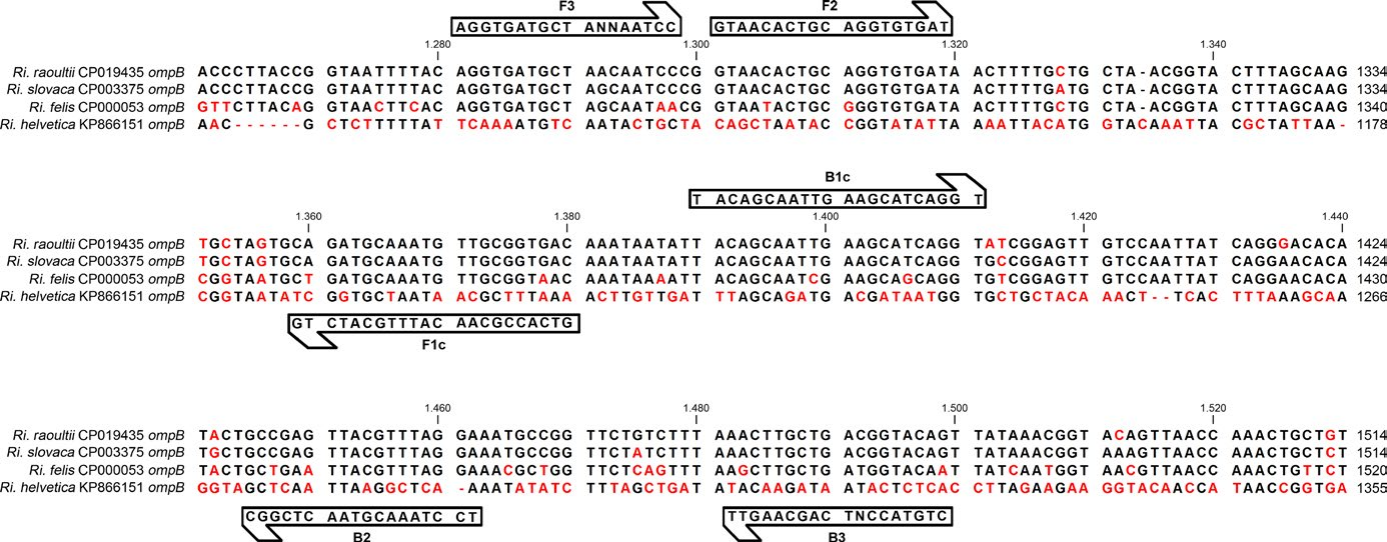
Schematic view of the *ompB* gene representing the target for the Rickettsia‐specific LAMP reaction. Arrow directions reflect the 5′‐3′ orientation of the oligonucleotides. Divergent nucleotides are in red

The LAMP approach confirmed positivity for both the *D. marginatus* PCR positive pools, while it did not return amplification products from any of the other pools. The comparison of the LAMP‐targeted gene *ompB* among the different species showed that the terminal region (roughly corresponding to the last 2,000 nucleotides of the coding sequence) in the four identified species was substantially conserved. Conversely, the gene significantly diverged in the region corresponding to the N‐terminus of the protein, with the exception of *Ri. raoultii* and *Ri. slovaca*, which share the 97% of their *ompB* gene nucleotide sequences. The analysis showed that the annealing regions of the LAMP primers, designed by Pan et al. (2012), fell within the most divergent portion of the gene, and therefore they did not match correctly in *Ri. felis* and *Ri. helvetica* (Figure 1 and Table 2). For these species, the divergence between primer and target sequences was not negligible, as they often mismatched within the last five nucleotides at the 3′ terminus.

### 3.4 Infection rate calculation

The calculated values of the infection rates are listed in Table 1. Considering all the identified ticks and *Rickettsia* species, the overall MLE was 26.19 per 1,000, with a 95% confidence interval ranging from 8.61 to 61.94 per 1,000. Considering the ticks species by species, MLE was 19.98 per 1,000, (95% confidence interval from 1.18 to 94.86 per 1,000) for *I. ricinus*, while it was 107.77 per 1,000 (95% confidence interval 21.11–330.80 per 1,000) for *D. marginatus*. Since only one pool was of *Rh. turanicus* in the study, it was not possible to calculate the MLE for that species.

## 4 DISCUSSION

Results from this study evidence the presence of four different SFG rickettsia species in ticks from Gargano National Park. They are in agreement with recent investigations in Southern Italy where, in addition to MSF, other TBR were reported from humans (Otranto et al., 2014).

Considering all tick species, the calculated IR was about 26 per 1,000, while taking into account *Ri. helvetica* and *I. ricinus* only, IR was about 20 per 1,000, quite lower if compared with the cited study and with environmental data from central Europe, reviewed by Karbowiak, Biernat, Stańczak, Szewczyk, and Werszko (2016). Conversely, the calculated IRs better agree with MIR reported by Severinsson, Jaenson, Pettersson, Falk, and Nilsson (2010), which ranged between 1.5 and 17.3 within ticks collected in Sweden. In anyway, even low infection rates of *Ri. helvetica* should not be disregarded, as the microorganism has been associated with perimyocarditis and meningitis, also leading to sudden death in humans (Portillo, Santibáñez, García Álvarez, Palomar, & Oteo, 2015).

On the other hand, infection rate was considerably higher for *D. marginatus* (107 per 1,000). Studies from Germany and Slovakia reported a prevalence of *Ri. slovaca* in *D. marginatus* and *D. reticulatus* up to 5%, and a prevalence up to 40.7% in Poland, and higher rates were recorded when *Ri. raoultii* is also considered (Karbowiak et al., 2016). In a previous investigation, 58.3% of tested *D. marginatus* specimens collected from humans were found positive in Southern Italy (Otranto et al., 2014), a rate consistent with data by Selmi et al. (2009), who reported high infection rate for this tick species.

*Rickettsia slovaca* and *Ri. raoultii* are causative agents of a syndrome known as DEBONEL/TIBOLA (Dermacentor‐borne necrosis erythema and lymphadenopathy/Tick‐borne lymphadenopathy) (Raoult et al., 2002). It is a newly recognized emerging disease, as its incidence has been increasing in Europe during the last decade (Portillo et al., 2015). Such trend is also confirmed in Italy, where the increasing detection of *Ri. slovaca* and *Ri. raoultii* counterweights the general decrease in traditionally MSF‐associated rickettsiae in both humans and ticks (Parola et al., 2009; Selmi, Bertolotti, Tomassone, & Mannelli, 2008).

It is not completely clear why circulation of *Ri. slovaca* and *Ri. raoulti* in wild environment is constantly increasing. It is tempting to hypothesize that their strong preference for *Dermacentor* spp. and, in turn, for wild boar, might provide a good substrate for the diffusion of the TIBOLA‐associated rickettsia species. In Apulia, wild boars were imported for repopulation purposes from Eastern Europe in the early 2000s, and they are now infesting many areas because of their high reproductive fitness. This may have contributed to the widespread diffusion of the two rickettsia species, together with the association of infected *D. marginatus* with some rodent species, as suggested by Martello et al. (2013). Unfortunately, no free‐ranging *D. marginatus* ticks were retrieved, so it was not possible to compare specimens from animal host with those from vegetation. This, along with the small sample size, makes not possible to confirm the hypotheses about the source of infection, but it may be a starting point to more clearly track the possible infection routes of *Ri. slovaca* and *Ri. raoultii*. In anyway, it is not negligible that the growing diffusion of those pathogens in ticks and in association with wild animals may increase the risk for humans to come in contact with infected hosts.

Finally, the only *Rh. turanicus* pool was found positive to *Ri. felis*, another emerging SFG‐rickettsia species. In humans, it causes a wide range of symptoms, including fever, myalgia, arthralgia, headache, abdominal pain, cough, chest pain, and febrile rash (Angelakis, Mediannikov, Parola, & Raoult, 2016); neurological symptoms were also reported (Zavala‐Velázquez, Ruiz‐Sosa, Sánchez‐Elias, Becerra‐Carmona, & Walker, 2000). Cat fleas are usually vectors and reservoirs of *Ri. felis* (Brown & Macaluso, 2016) but, more recently, the pathogen was also detected in several arthropods, including soft and hard ticks (Angelakis et al., 2016), the latter included in Rhipicephalinae subfamily (Abarca, López, Acosta‐Jamett, & Martínez‐Valdebenito, 2013). To the best of our knowledge, *Ri. felis* was never detected from *Rh. turanicus*, although it has been constantly recovered from fleas (Capelli et al., 2009; Persichetti et al., 2016). Therefore, although limited to only one positive pool, the finding of *Ri. felis* may be relevant in terms of public health. The infected tick was collected from a sheep, but, since no other *Ri. felis* was detected in this study, it is not possible to suggest a possible route for the transmission of such microorganism, from wild to domestic animals or vice versa. However, this finding may represent another proof of the increasing diffusion of *Ri. felis* in a very heterogeneous group of potential vectors.

In conclusion, considering the wide diffusion of rickettsiae and their arthropod hosts, there is a non‐negligible risk for people who live and work in proximity of infected or infested animals. Therefore, laboratory detection of SFG rickettsiae may be essential to recognize the pathogens in animals or vectors in the wild environment as well as in the clinical practice, in order to promptly diagnose, or even prevent, the infections in humans.

In this perspective, the LAMP approach for the detection of tick‐associated rickettsiae could be very useful, as it has the great advantage to be rapid and specific. LAMP is a promising technique, developed by Notomi et al. (2000) and briefly described in Raele, Pugliese, Galante, Latorre, and Cafiero (2016). In fact, it may return results in very short time, if compared with serological or PCR‐based techniques; the LAMP is also affordable for most of diagnostic laboratories (Raele et al., 2016), and it has already successfully been used for ticks endosymbionts (Raele, Galante, Pugliese, De Simone, & Cafiero, 2015).

However, the LAMP protocol used in this study failed to detect *Ri. helvetica* and *Ri. felis* in tick pools, while the PCR targeting the 17 kDa‐protein gene was more effective. This is probably linked to the variability in the *ompB* gene, target of this LAMP protocol. In fact, the designed oligonucleotides were found not to correctly match with the targets in *Ri. felis* and *Ri. helvetica,* presenting mismatches in the 3′ terminus. This leads to an unstable annealing of oligonucleotides that impedes the molecule to prime the polymerase activity.

Therefore, it should be strongly advisable to set up a new LAMP protocol based on more conserved region among *Rickettsia* genus, in order to gain a useful tool, which can greatly help the detection or diagnosis of those often neglected rickettsioses.

## CONFLICT OF INTEREST

None declared.
